# Does time heal all wounds? How is children’s exposure to intimate partner violence related to their current internalizing symptoms?

**DOI:** 10.3389/fpsyg.2022.998423

**Published:** 2022-11-03

**Authors:** Román Ronzón-Tirado, Natalia Redondo, María D. Zamarrón, Marina J. Muñoz Rivas

**Affiliations:** Department of Biological and Health Psychology, Autonomous University of Madrid, Madrid, Spain

**Keywords:** intimate partner violence exposure, internalizing symptoms, time, revicitmization, childhood maltreatment

## Abstract

The effects of time and the longitudinal course of the children’s internalizing symptoms following Intimate Partner Violence Exposure (IPVE) are still of great interest today. This study aimed to analyze the effect of the frequency of IPVE, adverse experiences after the cessation of the IPVE and the time elapsed since the termination of the violent relation on the prevalence of anxiety and depression among children. Participants were 107 children and their mothers who had been victims of IPV and had existing judicial protection and restraining orders. Hierarchical logistic regression models were estimated to analyze children’s adjustment, considering the effect of the time elapsed since the termination, frequency of IPVE, experiences of revictimization, maternal pathology, and anxious anticipation of the mother at the prospect of future harm. Exposure to multiple events of violence at the hands of multiple ex-partners and higher scores in the mother’s anxious anticipation were significant predictors of children’s pathological depression and anxiety. Our results emphasize the need for early psychological evaluation of women and children’s victims of IPV to provide timely interventions that avoid symptoms from becoming chronic. Strategies to bring support and emotional security to the victims after the end of the violent relationship are desirable.

## Introduction

Over the past few decades, a growing body of research has focused on analyzing the prevalence, risk factors, and consequences associated with exposure to intimate partner violence (IPV) among children aged 0 to 18 years old (Carlson et al., 2019; Van Eldik et al., 2020). Such research has shown that exposure to IPV, in itself, has negative consequences for children and can even be as harmful as direct abuse itself (Kitzmann et al., 2003; Castro et al., 2017). In general, the findings all indicate that the frequency with which children are exposed to instances of victimization inflicted on their mothers (especially psychological abuse) predicts the appearance and endurance of internalizing maladjustment processes (anxiety and depression; Vu et al., 2016; Carlson et al., 2019). However, there is some discrepancy among the different results regarding the factors associated with variability in the symptoms presented by children exposed to such situations, and findings tend to be inconclusive in terms of changes in the observed progression of clinical symptoms once exposure to such violence has ceased (Lauterbach and Armour, 2016; Carter et al., 2020).

The effects of the length of time and longitudinal course of the internalizing symptoms following exposure to IPV are still of great interest today, and three different theoretical approaches have been developed in this regard (Galano et al., 2021). According to theories of resilient response to trauma (Bonanno, 2004), the simple passage of time would be sufficient to improve children’s adjustment since the cessation of exposure to IPV could explain the decrease in internalizing symptoms. The effect of the passage of time would respond to a natural trend observed in people exposed to traumatic and violent events. Studies conducted from this perspective suggest that approximately 70% of children exposed would present mild initial clinical maladjustment that would disappear after exposure, 20% of children would recover within 4 to 12 months of exposure to trauma, returning to a mental state of health comparable to pre-exposure status within a maximum of 24 months, and only 10% of those affected would present chronic pathological indicators (Bonanno and Mancini, 2012; Galatzer-Levy et al., 2018; Meijer et al., 2019).

A second theoretical approach based on enduring effects models has suggested that the consequences of exposure to IPV among children would tend to remain stable regardless of the amount of time that has passed since exposure to IPV. According to these postulates, early socialization in violent experiences would be established as a relatively stable pattern of response in be behavior despite subsequent exposure to non-violent contexts (Fraley et al., 2013). Finally, a third approach, based on sleeper effect models, has suggested that internalizing symptoms would not be immediately apparent after exposure to abuse, but would be manifested later in development. In other words, children would initially tend to present subclinical indicators of maladjustment that would tend to increase and become chronic over time (Vu et al., 2016; Fong et al., 2019).

Despite the differences noted regarding the effect of the passage of time, the three previous theoretical perspectives all point to the existence of a group of children who consistently present internalizing symptoms even years after the cessation of exposure to violence (Kennedy et al., 2010; Van Eldik et al., 2020; Galano et al., 2021). Addressing the interaction of time and the cumulative value of exposure to different violent experiences that contribute to the development of pathological processes in children is still indispensable for the development of strategies focused on their treatment (D’Andrea and Graham-Berman, 2017; Jouriles et al., 2018; Carlson et al., 2019).

In order to obtain a more comprehensive view of the way children respond to exposure to IPV, it seems necessary to include in the analyses not only the cumulative value of past trauma and the passage of time itself (Bonanno, 2004; De La Vega et al., 2013; Castro et al., 2017; Berg et al., 2022), but also variables related to the context in which children develop once their mothers have separated from the aggressor that could affect children´ adjustment over time (Miller et al., 2012). In this regard, recent findings have emphasized that, in a manner similar to what the mothers have experienced, children’s exposure to IPV would take on different forms, resulting in continued experiences of revictimization (e.g., re-establishment of the mother-aggressor relationship and exposure to new violence events or exposure to multiple violent events at the hands of more than one partner against their mother) and stress sustained after the end of the violent relationship (e.g., maternal pathology or emotional insecurity associated with the mother’s anxious anticipation of potential future aggressions) likely to influence the endurance of pathological levels of anxiety and depression over time (Baird et al., 2021; D’Amore et al., 2021; Henze-Pedersen, 2022).

In this study, we draw on the conclusions reached in previous literature with the aim of identifying factors associated with the internalizing adjustment of children following exposure to IPV. We take variables pertaining to different levels of analysis, including not only the cumulative value of past trauma (De La Vega et al., 2013; Castro et al., 2017; Berg et al., 2022) but also other relational variables that could condition the mother–child relationship and the appropriate emotional support that the child would need after being exposed to IPV (e.g., experiences of revictimization, maternal pathology, and emotional security; Holmes, 2013; Jouriles et al., 2018; Silva et al., 2021). In the context of evaluating these research questions, we considered possible demographic covariates on children’s adjustment (D’Andrea and Graham-Berman, 2017; World Health Organization, 2018). Social variables such as economic stability, the mother’s level of education, and the family culture were measured and included in the analyses as covariates to analyze their possible effect on children’s level of well-being and health over time. Specifically, the three aims of this study were to: (a) examine the relationship between the time elapsed since the cessation of the IPVE on children’s anxiety and depression symptoms. (b) Analyze the cumulative effect of the frequency of IPVE and children’s adverse experiences after the termination of the violent relation on the presence/absence of anxious-depressive symptoms. (c) Analyze the effect of the children’s adverse experiences after the termination of the violent relationship on the relation between the time elapsed since the cessation of the IPVE on children’s anxiety and depression symptoms.

## Materials and methods

### Procedure

This study was developed in collaboration with the Spanish Home Office Department of Security, responsible for the VioGén National Integrated Monitoring System for Gender-Based Violence. Police officers from the 21 districts of Madrid were initially asked to inform IPV victims with active judicial measures associated with intimate partner violence and registered in the VioGen system about the possibility of collaborating in a study on the consequences associated with IPV victimization among women and the children in their charge. A total of 1765 women were contacted, 567 agreed to participate (32% of the total). The women who gave their consent to the police officers were then contacted by members of the research team by telephone to set up a date for the interview. At his point 40% of the women decided not to continue in the study. The remaining 338 were interviewed by the licensed psychologist trained by the research team on IPV victim’s assistance. The interviews lasted approximately 2 hours and were conducted at the victims’ homes or at the police station in charge of providing the victims with judicial protection. According to the aims of this study, only the victims with at least one child (*n* = 107) were included in the analyses. The procedure was approved by the [masked for review process] Ethics Committee.

### Participants

The study included 107 children and their mothers who had been victims of intimate partner violence and who had existing judicial protection and restraining orders taken out after they reported intimate partner violence registered in the Spain’s Integrated Monitoring System in cases of Gender Violence (VioGén System), filed with the Spanish Department of Security. The children were aged between 6 and 12 years old (*M* = 9.41; *SD* = 2.04); 52.5% were girls and 47.5% were boys. 30.3% were only children and the remaining 69.7% had siblings (34.2% one sibling, 22.4% two siblings, 13.2% three siblings or more). All children were in primary education. All the children lived with their mothers. 33.8% of the mothers reported having a monthly income of less than €600, 25.8% between €601 and €1,000, 22.5% between €1,001 and €1,500, and 17.7% higher than €1500.60.5% of the mothers were Spanish and the remaining 39.5% were foreign nationals (33.5% Latinos and 3.9% Eastern Europe). Around 19.3% of the mothers had a primary level of education, 43.4% a secondary or high school education, 21.7% vocational training, and 15.7% university or postgraduate studies.

### Group assignment

Participants were divided into three mutually exclusive groups: (1) children of women who had been victims of one IPV episode and had a single IPV police report since 2014 (VSR), (2) Children of women who had been victims of IPV and had felt more than one police report against the same aggressor (Victims with multiple police reports by the same aggressor; VMRSA), and (3) Children of women who had been victims of IPV and had felt police reports against two or more aggressors (Victims with multiple police reports by the multiple aggressors; VMRMA). Group assignment was based on VioGen data records and women’s interviews about IPV history of abuse. Around 36.8% of children were the children of Victims with a single report (VSR), 43.4% were the children of Victims with multiple reports by the same aggressor (VMRSA), and the remaining 19.8% were the children of victims with multiple police reports by the multiple aggressors (VMRMA).

### Measurements

#### Sociodemographic and relationship data

Multiple option items were used to collect sociodemographic about children’s and their mother’s age, gender monthly income, and nationality, as well as data related to the children’s exposure to violence such as the typology of exposure to IPV (by a single aggressor or multiple aggressors), the number of complaints filed by the mother associated with gender-based violence, the presence/absence of direct maltreatment of the children by the ex-partner, and the mother’s anticipatory anxiety (fear) regarding the possibility that her ex-partner(s) might assault her or her children again.

#### Children’s pathology

The Spanish adaptation of (Sardinero García et al., 1997) of the Child Behavior Checklist 6–18 (CBCL, Achenbach, 1991) was used to measure children’s internalized symptoms. It consists of 118 items scored by parents describing externalizing and internalizing problems of their children (6 to 18 years of age). The statements have three response options ranging from 0 to 2 (0 = *does not occur*, 1 = s*ometimes*, 2 = *very often*). For the purposes of this study, only the dimensions corresponding to anxiety (13 items; e.g. “*is afraid of certain situations, animals or things*,” “*cries a lot*,” “*feel they have to be perfect*,” “*nervous, tense*,” “*gets self-conscious or embarrassed easily*”) and depression (8 items; e.g. “*enjoys very few things*,” “*prefers to be alone than with other people*,” “*refuses to talk*,” “*not very active, slow or lacking energy*”) were used. The scores obtained for each scale were transformed to T scores, T values higher than 65 were considered pathological (Unitat d'Epidemiologia i de Diagnóstic en Psicopatologia del Desenvolupament-UAB- y Servicio de Psicología Aplicada-UNED, 2010), the reliability indices obtained for each dimension have been α =0.82 [0.75–0.87] for anxiety and α =0.72 [0.63–0.80] for depression.

#### Intimate partner violence exposure

IPVE was measured through The Revised Conflict Tactics Scales (CTS2; Straus et al., 1996). It is a Likert-type scale with 8 response options (0 = *never occurred*, 1 = *once*, 2 = *twice*, 3 = *three to five times*, 4 = *6 to 10 times*, 5 = *11 to 20 times*, 6 = *more than 20 times*, 7 = *not in the last year but before*) to score the frequency of violent behaviors within intimate relationships during the last year. The scale is composed of 8 dimensions, 4 on perpetration and 4 on victimization. For the purposes of this study, two of the victimization scales were used: psychological victimization and physical victimization. Mothers were asked about the type and frequency of exposure of children to each type of aggression during the last year of living with the last aggressor. The reliability indices obtained for each dimension were α =0.87 [0.82–0.91] for verbal victimization; α =0.93 [0.90–0.95] for physical victimization.

***Time since the separation from the aggressor*** was calculated accounting for the time elapsed since the date on which the last restraining order was established and the date when the interview took place.

#### Children’s adverse experiences after the termination of the violent relationship

(1) *Mothers’ pathology* was measured based on the total score of the Spanish adaptation (Daza et al., 2002) of the Depression Anxiety Stress Scales (DASS: Lovibond and Lovibond, 1995). The DASS is set of three self-report scales designed to measure the emotional states of depression, anxiety, and stress, each dimension of the DASS is composed of 7 Likert-type items with response options from 0 to 3 (0 = “*not at all*,” 1 = “*a little*,” 2 = “*quite a lot*,” 3 = “*a lot*”). The depression dimension assesses dysphoria, lack of interest, anhedonia, and self-depreciation. The anxiety dimension assesses autonomic activation, situational anxiety, and subjective experience of anxious affect. The stress dimension assesses persistent non-specific activation, irritability and difficulty relaxing. The reliability indices obtained for each dimension were α =0.74 [0.65–0.82] for anxiety; α =. 92[0.85–0.94] for depression and α =0.72 [0.63–0.81] for stress. (2) Mothers’ anticipatory anxiety about future harm was assessed through two Likert-type items (e.g.*, “How afraid are you that the person you have reported may harm you again?,” “How afraid are you that the person you have reported may harm your children?”*) with response options from 0 to 10 (1 = *not afraid at all*, 10 = *extreme fear*), both items had a correlation equal to *r* = 0.67, *p* < 0.001. As suggested by prior DASS validation studies in Spanish-speaking samples (Román et al., 2016), cut-off scores for the depression scale was 6 and above, 5 and above for anxiety, and 6 and above for stress.

### Data analysis

Firstly, the internal consistency of the scales used in the study was analyzed using the Cronbach Alpha coefficient. Coefficients greater than.60 were considered acceptable (Taber, 2018). Subsequently, the assumption of normality of distribution for the depression and anxiety scores of the children was contracted using the K-S test. Descriptive statistics and the distribution of frequencies were analyzed for the symptoms presented by the children, sociodemographic data, and characteristics of the history of exposure to intimate partner violence. T-tests were performed to analyze differences in the number of months since the cessation of IPVE and the presence/absence of clinical symptomatology in children (i.e., depression and anxiety). Pearson’s *X^2^* tests were used to contrast the differences in the context variables and in relation to exposure to violence. Subsequently, hierarchical logistic regression models were estimated in order to predict children’s adjustment based on the effect of the time elapsed since the end of the violent relation, the frequency of the IPVE, and the children’s adverse experiences after the termination of the violent relation. Finally, interaction terms were tested for the effect of the time elapsed since the IPVE and the each of the children’s adverse experiences after the termination of the violent relation (i.e., experiences of revictimization by the same or multiple aggressors, mothers pathology and mothers anticipatory anxiety about future harm) each interaction term was entered in different steps. All analyses were performed using the SPSS statistical software version 25. We dealt missing data with listwise deletion method in all our analysis. In terms of efficiency and consistency the small percentage of missing data (3.5%) suggest results are unbiased. Complete case analysis with less than 5% of missing data are recommended (Drechsler, 2015).

## Results

Over 19.8% of children had been exposed to IPV multiple times at the hands of more than one of their mother’s ex-partners, 43.4% of children had been exposed to IPV multiple times at the hands of a single ex-partner, and the remaining 36.8% had been exposed to IPV by a single aggressor. Around 40% of the children had suffered direct abuse at the hands of their mother’s last ex-partner (Table 1). Around 53.8% of children exposed to IPV maintained pathological levels of anxiety or depression, 15.4% had anxiety, 14.4% had depression, and the remaining 24% had both anxiety and depression.

**Table 1:** Descriptive statistics of study variables.

	*n*	%	Mean	SD	Range
*Children’s pathology*					
Anxiety CBCL	40	38.5	6.06	4.73	23
Depression CBCL	43	40.6	3.61	2.68	12
*Mother’s pathology*					
Stress DASS	78	78.0	9.83	7.88	51
Anxiety DASS	75	72.1	7.97	7.69	49
Depression DASS	49	47.6	6.90	6.12	20
*IPV Exposure*					
Direct maltreatment^a^	32	40.0	–	–	
Psychological IPV			107.97	56.75	194
Physical IPV			70.20	77.16	283
Anticipating future damages	–	–	10.24	7.01	18
Time elapsed	–	–	27.45	24.12	84
Type of victim					
VSR	39	36.8	–	–	–
VMRSA	46	43.4	–	–	–
VMRDA	21	19.8	–	–	–

^a^ = Childhood maltreatment exerted by the ex-partner; Time elapsed = Time elapsed since the separation; CBCL = Child Behaviour Check List. DASS = Depression Anxiety Stress Scales. VSR = Victim with a Single Report; VMRSA = Victim of multiple reports by the same aggressor; VMRDA = Victims of multiple reports by diverse aggressors. ** *p* < 0.01; * *p* < 0.05.

The time passed since the establishment of a restraining order associated with the last report of intimate partner violence ranged from 1 to 84 months (*M* = 27.45; *SD* = 24.12). A greater number of months was associated with lower levels of anxiety (*t* = 2.687; *p = 0.*005) and depression (*t* = 2.20; *p = 0.*030). However, around 36% of children for whom between two and 3 years had passed since the end of the violent relationship continued to maintain pathological levels of internalizing symptoms (35.9% anxiety and 37.5% depression), and around 20% of children for whom more than 3 years had passed since separation from the violent relationship continued to maintain pathological levels of anxiety and depression (20 and 24%, respectively; Table 2).

**Table 2:** Differences in children’s anxiety and depression related to sociodemographic/contextual and IPVE variables.

	*Categories*	Anxiety					Depression				
	*No pathologicn %*		*Pathologicn %*		*X^2^*	*No pathologicn %*		*Pathologicn %*		*X^2^*
*Sociodemographic/contextual*											
Gender	Boy Girls	29 34	61.2 61.8	22 21	38.8 38.2	0.01	29 34	61.2 61.8	22 21	38.8 38.2	0.08
Child age	6–9 10–12	20 27	57.2 60.0	15 18	42.9 40.0	0.07	18 28	51.4 62.2	17 17	48.6 37.8	0.93
Migrant	No Yes	36 28	64.3 58.3	20 20	35.7 41.7	0.387	30 33	53.6 67.3	26 16	46.4 32.7	2.07
Social assistance	No Yes	43 20	61.4 64.5	27 11	38.6 35.5	2.34	40 20	56.3 64.5	31 11	43.7 35.5	1.98
Mother’s income	≤600 601–1,000 1,001–1,500 >1,500	23 16 8 10	67.6 48.5 50.0 83.3	11 17 8 2	32.4 51.5 50.0 16.7	6.04	19 19 9 7	55.9 57.6 52.9 58.3	15 14 8 5	44.1 42.4 47.1 41.7	0.122
Mother’s level of education	Elementary Secondary VE University	15 24 16 9	71.4 57.1 66.7 52.9	6 18 8 8	28.6 42.9 33.3 47.1	2.01	14 24 18 7	66.7 57.1 75.0 41.2	7 18 6 10	33.3 42.9 25.0 58.8	5.30
*IPVE related*											
Time elapsed since last IPV complaint	≤1 year 2–3 years >3 years	19 25 20	47.5 64.1 80.0	21 14 5	52.5 35.9 20.0	7.04*	19 25 19	46.3 62.5 76.0	22 15 25	53.7 37.5 24.0	5.92*
Type of victim	VSR VMRSA VMRDA	26 30 8	66.7 68.2 38.1	13 14 13	33.3 31.8 61.9	6.13*	28 27 8	71.8 58.7 38.1	11 19 13	28.2 41.3 61.9	6.49*
Childhood maltreatment	No Yes	10 42	71.4 62.7	4 25	28.6 37.3	0.385	10 40	71.4 59.7	4 27	28.6 40.3	0.674
Anxious anticipation of future damages	Low (0–6) Medium (7–13) Height (14–20)	30 13 15	76.9 72.2 45.5	9 5 18	23.1 27.8 54.5	8.32*	30 10 15	76.9 55.6 45.5	9 8 18	23.1 44.4 54.5	7.74*
Mother’s clinical anxiety	No Yes	26 38	89.7 50.7	8 37	10.3 49.3	13.4***	20 43	69.0 57.3	9 32	31.0 42.7	1.18
Mothers’ clinical depression	No Yes	15 46	68.2 59.0	7 32	31.8 41.0	0.61	15 44	68.2 56.4	7 34	31.8 43.9	0.98
Mothers clinical stress	No Yes	24 39	49.0 72.2	25 15	51.0 27.8	5.84*	28 34	51.7 63.0	21 20	42.9 37.0	0.36

IPV = Intimate Partner Violence Exposure; VE = Vocational education; VSR = Victim with a Single Report; VMRSA = Victim of multiple reports by the same aggressor; VMRDA = Victims of multiple reports by diverse aggressors; ^a^ Childhood maltreatment exerted by the ex-partner of the mother. *** *p* < 0.001; * *p* < 0.05.

Analysis regarding differences in sociodemographic/contextual and trauma-related variables associated with the internalizing adjustment of the children indicated that none of the variables suggested in previous studies could discriminate group membership with pathological anxiety or depression (Table 2). However, comparisons with variables related to the trauma experienced by children allowed us to identify that both pathological anxiety and depression among children were related to experiences of sustained stress, specifically, when the mothers suffer episodes of IPV by new aggressors after a first incident (*X^2^* = 6.13; *p* < 0.05; *AR*_ji_ *=* 2.5; for anxiety and 6.49; *p* < 0.05; *AR*_ji_ *=* 2.2; for depression). Likewise, higher scores for maternal anxious anticipation were associated with greater pathology among the children (*t* = −2.81; *p* < 0.01 for anxiety and − 2.15; *p* < 0.05; for depression). Finally, the analyses showed that maternal pathology was only related to pathological anxiety in children (pathological anxiety of the mother *X^2^* = 13.4; *p* < 0.001; pathological stress of the mother *X^2^* = 5.84; *p* < 0.05) and not to depression in children.

### Hierarchical logistic regression models for pathological anxiety and depression

The hierarchical logistic regression model explained 56% of the variance observed in relation to the development of pathological anxiety in children exposed to IPV (see Table 3). The number of months that passed since the establishment of a restraining and legal protection order was shown to be a significant predictor of child improvement despite differences in the frequency of exposure to psychological and physical IPV [Exp(B) = 0.976, *p* = 0.048; Step 1]. However, when including the effect of revictimization, anxious anticipation about potential further IPV, and maternal pathology (Step 2), we found that time itself was no longer significant and instead, the relevant predictors were: exposure to multiple events of violence at the hands of multiple ex-partners {as compared to exposure to a single event [Exp(B) = 0.098, *p* = 0.011] or multiple events of violence at the hands of the same ex-partner [Exp(B) = 0.096, *p* = 0.008]}, as well as higher scores in the mother’s anxious anticipation regarding future abuse [Exp(B) = 1,175, *p* = 0.005]. Finally (Step 3), we analyzed the interaction effect of time with different types of revictimization and anxious anticipation on the part of the mother. The results pointed to a significant interaction between time elapsed since the termination and the type of revictimization such that a greater number of months predicted improvement in children exposed to IPV by a single abuser on one occasion [Exp(B) = −0.175, *p* = 0.028] and on multiple occasions [Exp(B) = −0.068, *p* = 0.050], but not in the case of children exposed to IPV by multiple aggressors. The interaction between time and anxious anticipation was not significant, suggesting that the time elapsed since violence cessation would not affect the relationship between the anxious anticipation of the mother and the pathology of the children (Step 4).

**Table 3:** Hierarchical logistic regression models predicting children’s pathological anxiety after IPV exposure.

	Step 1			Step 2			Step 3 Interaction term		
	*β*	*SE*	Exp(B) [CI]	*β*	*SE*	Exp(B)	*β*	*SE*	Exp(B) [CI]
Time elapsed	−0.024*	0.013	0.976[0.95–1.01]	−0.022	0.014	0.978[0.951–1.01]	0.029	0.027	1.03[0.98–1.08]
IPVE psychological	0.011	0.006	1.01[0.99–1.02]	0.011	0.008	1.01[0.99–1.02]	0.018*	0.009	1.01[1.00–1.03]
IPVE physical	0.008	0.005	0.992[0.98–1.00]	−0.13	0.007	0.979[0.96–0.99]	−0.014	0.008	0.978[0.96–0.99]
Type of victim^a^									
VSR				−2.32*	0.909	0.099[0.02–0.58]	1.00	1.49	2.72[0.14–5.61]
VMRSA				−2.35**	0.890	0.096[0.02–0.55]	−0.756	1.17	0.47[0.04–4.7]
Anticipating future damages/				0.161**	0.057	1.18[0.98–1.09]	0.175**	0.066	1.19[1.04–1.35]
Mothers’ pathology				0.036	0.026	1.03[0.98–1.09]	0.024	0.027	1.02[0.97–1.08]
Time*Type of victim									
Time*VSR							−0.175*	0.080	0.839[0.71–0.98]
Time*VMRSA							−0.068*	0.036	0.934[0.87–1.01]
Constant	−0.665	0.645	0.51	−0.334	1.05	0.716	−2.387	1.42	0.092
*R^2 Nagelkerke^*			0.153			0.465			0.563
*−2 Log likehood*			91.47			68.55			59.75
Δ*R^2^*			0.153***			0.312***			0.098*

Time elapsed since the last complaint and establishment of the restriction order; ^a^ = Reference category is VMRDA; IPVE = Intimate Partner Violence Exposure Frequency; VSR = Victim with a Single Report; VMRSA = Victim of multiple reports by the same aggressor; VMRDA = Victims of multiple reports by diverse aggressors; Mothers ‘pathology refers to the presence of clinical levels of depression or anxiety in the mothers. *** *p* < 0.001; ** *p* < 0.01; * *p* < 0.05

The hierarchical logistic regression model developed for depressive pathology (Table 4), on the other hand, explained 35% of the variance observed. The results showed that the time elapsed since violence cessation was not related to the decrease in the pathology of children, when controlling for the frequency of exposure to IPV (Step 1) and trauma-related variables (Step 2). The variables that significantly predicted depressive pathology were: higher frequency of exposure to psychological IPV [Exp(B) = 1.02; *p* = 0.005]; having been exposed to multiple IPV events by different ex-partners {compared to exposure to a single event [Exp(B) = 0.21, *p* = 0.048] or multiple violent events at the hands of a single partner [Exp(B) = 0.017, *p* = 0.021]}; and higher scores in anxious anticipation of future damages on the part of the mother [Exp(B) = 1.10; *p* = 0.033]. The interaction effects of time*type of victimization and time*anxious anticipation (Step 3, Step 4) were not significant, thus model 2 was selected as the best-fitting model for the data.

**Table 4:** Hierarchical logistic regression models predicting children’s pathological depression after IPV exposure.

	Step 1			Step 2			Step 3 Interaction term		
	*β*	*SE*	Exp(B) [CI]	*β*	*SE*	Exp(B)	*β*	*SE*	Exp(B) [CI]
Time elapsed	−0.019	0.011	0.98[0.96–1.01]	−0.017	0.013	0.983[0.95–1.01]	0.017	0.025	1.01[0.96–1.06]
IPVE psychological	0.017*	0.007	1.01[1.00–1.03]	0.017*	0.007	1.02[1.00–1.03]	0.020*	0.008	1.02[1.01–1.03]
IPVE physical	−0.003	0.004	0.997[0.98–1.01]	−0.007	0.005	0.99[0.98–1.00]	−0.007	0.005	0.993[0.983–1.01]
Type of victim^a^									
VSR				−1.54*	0.781	0.21[0.05–0.98]	−0.397	1.16	0.672[0.07–6.6]
VMRSA				−1.79*	0.776	0.17[0.03–0.76]	−0.751	1.09	0.472[0.05–4.0]
Anticipating future damages/				0.101*	0.047	1.11[1.00–1.21]	0.102*	0.048	1.10[1.01–1.21]
Mothers’ pathology				−0.005	0.024	0.99[0.95–1.04]	−0.005	0.024	0.99[0.94–1.04]
Time*Type of victim									
Time*VSR							−0.045	0.034	0.95[0.89–1.02]
Time*VMRSA							−0.044	0.032	0.95[0.89–1.02]
Constant	−1.64*	0.706	0.192	−1.15	0.99	0.315	−2.35	1.318	0.095
*R^2 Nagelkerke^*			0.187			0.330			0.357
*−2 Log likehood*			92.41			82.24			80.12
Δ*R^2^*			0.187***			0.138**			0.027

Time elapsed since the last complain and establishment of the restriction order; ^a^ = Reference category is polyvicitm; IPVE = Intimate Partner Violence Exposure Frequency; VSR = Victim with a Single Report; VMRSA = Victim of multiple reports by the same aggressor; VMRDA = Victims of multiple reports by diverse aggressors. Mothers ‘pathology refers to the presence of clinical levels of depression or anxiety in the mothers. *** *p* < 0.001; ** *p* < 0.01; * *p* < 0.05.

## Discussion

The study presented here is the first analysis developed in the Spanish population of children recognized as victims of violence by the Spanish State, as the children of women victims of intimate partner violence registered in the VioGen system. Our study identified the high prevalence of anxiety and depression in children exposed to IPV. More than half of the children presented symptoms of depression, anxiety, or both to a clinically significant degree. In the study sample, the findings corroborated that the consequences suffered by children were similar to those presented by other samples exposed to direct physical or sexual abuse (Amado et al., 2015; Castro et al., 2017) and higher than those presented by children exposed to other traumatic and violent events such as community violence, peer violence, or accidents (Galatzer-Levy et al., 2018; Jadambaa et al., 2020). In addition, our findings make an important contribution to the study of this problem, by documenting that many of the children exposed to IPV continued to present pathological levels of anxiety and depression even after three or more years had passed since the end of the violent relationship and they were still protected by active judicial measures. Contrary to the theories of resilient response to trauma (Bonanno and Mancini, 2012; Galatzer-Levy et al., 2018; Meijer et al., 2019), our findings have shown that, after exposure to IPV, more than 50% of children had pathological symptoms of anxiety and depression and, after 24 months, about 30% of children continued to present such symptoms. Indeed, our findings seem to partly corroborate the proposals put forward by lasting effect models and sleeper effect models, which indicate that symptoms become chronic with the passing of time, associated with early socialization in violent experiences, and that symptoms crystallize over time associated with the stages of development in children (Vu et al., 2016). Further studies based on longitudinal data are recommended to analyze the course of the symptoms as well as other potentially stressful situations that children may experience after the termination of the violent relationship.

Overall, the results suggest that exposure to IPV in itself represents a continuing unease over time with significant long-term consequences, where simple separation from the aggressor does not necessarily facilitate an improvement in the child’s well-being. The findings suggest that the adjustment and pathology of these children are conditioned by a complex process, influenced by the effect of revictimization experiences and stress sustained even after the mother has ended her violent relationship (Hayes, 2017; Baird et al., 2021; Henze-Pedersen, 2022). In this sense, one of the main contributions of the study has been to note that the endurance of high levels of maladjustment over time is not only characteristic of children seen in shelters or by social service agencies following exposure to IPV who may be affected by other additional elements of stress that would justify this (e.g., separation from the home) but can also be observed in children who remain in their homes in a relatively stable context (Jouriles et al., 2014; Graham-Bermann and Miller-Graff, 2015).

Specifically, the results suggest that pathological depression in children exposed to IPV, unlike anxiety, is more stable and less sensitive to the time elapsed since the termination of the violent relationship. As in the previous literature, depressive symptomatology has been associated with a previous history of victimization, the effects of which tend to remain stable despite changes in the context surrounding the child (Kennedy et al., 2010; Silva et al., 2021). Thus, more frequent exposure to psychological IPV prior to the separation has been associated with a greater presence of clinical depression. Anxiety, on the other hand, is found to be more sensitive to the time elapsed since violence exposure cessation, as well as to changes in the context surrounding the child associated with the process of separation and termination of the violent relationship. In this regard, the results indicate that a time-lapse of a greater number of months since the end of the violent relationship is associated with a lower presence of clinical anxiety symptoms. Experiences of stress sustained after separation are significantly related to a greater probability of maladjustment in the child (Meijer et al., 2019). In addition, as an additional explanation to the increased salience of depressive symptoms in children exposed to IPV, future studies should analyze the possible precedence of anxiety that is not treated and managed through non-adaptive strategies such as avoidance. This process has been documented among children exposed to other traumatic events (e.g., natural disasters; Cheng et al., 2020).

Regarding the similarities in the course of both pathologies, our findings corroborate the invariance of the impact of IPV exposure according to the sex and age of the children (Davies et al., 2016). It has also been identified that repeated experiences of IPV exposure were related to a higher prevalence of depression and anxiety. Thus, children exposed to multiple events of IPV at the hands of different former partners of their mothers were the ones who on average were most likely to suffer and maintain both anxiety and clinical depression. This relationship between revictimization and pathology, according to the previous literature, would partly respond to the cumulative value of the aggressions witnessed (Rutter, 1985; De La Vega et al., 2013; Castro et al., 2017), where a greater number of victimization experiences would be associated with poorer adjustment. However, future studies should return to this finding and analyze it based on changes in the mother–child relationship. It is possible that children exposed to multiple events of IPV at the hands of different aggressors tend to feel more insecure about the surrounding context, making it difficult to adjust later even after their mother has separated from the aggressor. In light of these results, we should emphasize the importance for the current knowledge on intimate partner violence of the distinction made in this study about the different types of victimization in relation to children. Thus, distinguishing between single episodes of violence, multiple episodes by a single aggressor, and multiple episodes by different aggressors has made it possible to establish different psychological consequences for children, and they are most severe among children who have been exposed to revictimization by multiple aggressors.

Finally, with regard to the stress sustained after separation, we identified that high levels of anxious anticipation in the mother about future abuse were related to a higher prevalence of depression and anxiety among the children. This finding corroborates the proven relationship between maternal well-being and child adjustment (Holmes, 2013; Jouriles et al., 2018; Silva et al., 2021). Contrary to the hypothesis, our results point out that the pathology of the mother is not related to the child’s maladjustment. However, these results must be interpreted with caution given the high prevalence of pathology in the mothers (E.g. as in previous studies we identified that most women reported clinical symptoms; Jouriles et al., 2018). We would recommend continuing to develop intervention strategies focused on both the clinical and emotional state of the mothers and their children and the mother–child relationship after separation from the violent relationship (Graham-Bermann and Miller-Graff, 2015; Galano et al., 2021), paying particular attention to the cognitive assessment of both of them with regard to perceived current risk, as well as the emotional bond and security (mother–child/ren) following the process of separation/re-establishment and eventual termination of the violent relationship. Furthermore, these findings support previous studies (Henze-Pedersen, 2022), in terms of the need to recognize how violence and memories of violence are experienced by women and their children after IPV cessation, as well as the need to un-blame mothers for their own symptoms of depression and anxiety and the consequences these might have on their children’s adjustment. That is, to recognize that mothers’ and children’s symptoms are primarily consequences of a severe trauma and the harm inflicted by the perpetrator.

The following limitations should be considered when analyzing the results and conclusions presented in this study: First, a cross-sectional design has been used in this study, making it difficult to follow up on time and changes in the trajectory of the children’s symptoms. Therefore, interpretations based on our results should be limited to the prediction the children symptoms at the time of study based on time since separation from the abuser; Second, although one of the main contributions of this study is that it is the first analysis carried out in relation to the Spanish VioGen system, results may not be generalizable since sampling was limited to women with a history of reported IPV and active restraining orders in Madrid, Spain. Third, given the sample only includes mothers/children in the VioGen system, there was not comparison group to establish the counterfactual of child wellbeing outcomes. We might expect the mothers/children who got the restraining order to fare better over time than mothers/children who did not. Additionally, data about stalking or abuse post-separation was not gathered or controlled in our analyses, further studies on the cumulative effect of victimization experiences (i.e., perpetrated by the ex-partner, family, institutions) after relationship termination are desirable. Fourth, children’s self-reports of violence exposure and mental health were not obtained, and scores on children’s symptoms may be biased depending on mother’s view, further studies should aim to overlap scores by multi-informants. Finally, when categorizing continuous variables (i.e., depression, anxiety), to consider the presence/absence of pathology, it is possible that the analyses may have lost statistical power. Therefore, future research should examine the relationships addressed in this study using continuous measures.

However, the present study has some important clinical implications. First of all, it emphasizes the need for early psychological evaluation of women and children identified as victims of intimate partner violence in order to provide timely interventions that avoid symptoms becoming chronic and prevent exposure to new incidents of IPV committed against the mothers. The results of this study show how early intervention is especially valuable because, although the number of months elapsed since the termination of the violent relationship seem to bring an improvement in the anxious symptoms of the children, this advantage disappears when new incidents of IPV occur or when the mother anticipates the possibility of them. Secondly, it underlines the need for a continuous evaluation over time of the symptomatology of the victims (women-children) that allows for a deeper understanding of the mechanisms of adjustment subsequent to separation from the aggressor and the expected trajectories of pathology in both mother and child(ren). Thirdly, the results indicate the impact that police and judicial measures have on the emotional development of children, showing how restraining orders predict an improvement in anxious symptoms among children regardless of the intensity of exposure to violence. Finally, it corroborates the need to establish specific intervention programmes and other government measures that focus their efforts on establishing a climate of support and emotional security after the end of the violent relationship (D’Andrea and Graham-Berman, 2017; Jouriles et al., 2018; Galano et al., 2021).

## Data availability statement

The datasets presented in this article are not readily available because data are available on reasonable request and on signature of a confidentiality agreement from author MM. Requests to access the datasets should be directed to marina.munoz@uam.es.

## Ethics statement

The studies involving human participants were reviewed and approved by the procurement of the data required for this study by the Autonomous University of Madrid ethics review board. All procedures performed in this study were in accordance with the ethical standards of the university ethics committee and with the 1964 Helsinki declaration and its later amendments or comparable ethical standards. Written informed consent to participate in this study was provided by the participants’ legal guardian/next of kin.

## Author contributions

RR-T, NR, MZ, and MM-R contributed with the conceptualization, investigation, formal analysis, and writing. All authors contributed to the article and approved the submitted version.

## Funding

This work has been funded by a research grant from the Ministry of Science, Innovation and Universities of Spain (PGC2018-096130-B-I00). And the predoctoral contract (PRE2019-091184).

## Conflict of interest

The authors declare that the research was conducted in the absence of any commercial or financial relationships that could be construed as a potential conflict of interest.

## Publisher’s note

All claims expressed in this article are solely those of the authors and do not necessarily represent those of their affiliated organizations, or those of the publisher, the editors and the reviewers. Any product that may be evaluated in this article, or claim that may be made by its manufacturer, is not guaranteed or endorsed by the publisher.
